# Derivatization-free gel permeation chromatography elucidates enzymatic cellulose hydrolysis

**DOI:** 10.1186/1754-6834-5-77

**Published:** 2012-10-12

**Authors:** Philip Engel, Lea Hein, Antje C Spiess

**Affiliations:** 1 AVT-Enzyme Process Technology, RWTH Aachen University, Worringerweg 1, Aachen, 52056, Germany; 2 Interactive Materials Research, DWI an der RWTH Aachen e.V, Forckenbeckstr. 50, Aachen, 52074, Germany

**Keywords:** Cellulose molecular weight, Gel permeation chromatography, Eluent for underivatized cellulose

## Abstract

**Background:**

The analysis of cellulose molecular weight distributions by gel permeation chromatography (GPC) is a powerful tool to obtain detailed information on enzymatic cellulose hydrolysis, supporting the development of economically viable biorefinery processes. Unfortunately, due to work and time consuming sample preparation, the measurement of cellulose molecular weight distributions has a limited applicability until now.

**Results:**

In this work we present a new method to analyze cellulose molecular weight distributions that does not require any prior cellulose swelling, activation, or derivatization. The cellulose samples were directly dissolved in dimethylformamide (DMF) containing 10-20% (v/v) 1-ethyl-3-methylimidazolium acetate (EMIM Ac) for 60 minutes, thereby reducing the sample preparation time from several days to a few hours. The samples were filtrated 0.2 μm to avoid column blocking, separated at 0.5 mL/min using hydrophilic separation media and were detected using differential refractive index/multi angle laser light scattering (dRI/MALLS). The applicability of this method was evaluated for the three cellulose types Avicel, α-cellulose and Sigmacell. Afterwards, this method was used to measure the changes in molecular weight distributions during the enzymatic hydrolysis of the different untreated and ionic liquid pretreated cellulose substrates. The molecular weight distributions showed a stronger shift to smaller molecular weights during enzymatic hydrolysis using a commercial cellulase preparation for cellulose with lower crystallinity. This was even more pronounced for ionic liquid-pretreated cellulose.

**Conclusions:**

In conclusion, this strongly simplified GPC method for cellulose molecular weight distribution allowed for the first time to demonstrate the influence of cellulose properties and pretreatment on the mode of enzymatic hydrolysis.

## Background

Gel permeation chromatography is a well-established technology to provide in-depth information on the cellulose polymer molecular weight distribution [1]. This can be particularly useful to understand and improve the enzymatic cellulose hydrolysis which is an essential aspect for efficient biomass utilization [2]. Until now cellulose pretreatment and enzymatic hydrolysis efficiency is primarily assessed by soluble sugar analysis [3,4] that is evaluated and correlated to the corresponding substrate properties of cellulose: crystallinity, particle size, and accessible surface area [5-7]. However, the focus on sugar formation excludes substantial aspects of the hydrolysis reaction: The enzymatic cellulose hydrolysis is performed by a mixture of different endo- and exo-acting enzymes. While exoglucanases cleave soluble cellobiose from the cellulose polymer, the endoglucanases cut the polymer in the interior of the chain, not necessarily resulting in direct soluble sugar formation [8]. The statistical release of shorter polymer products would provide information about hydrolysis and the effect of pretreatment efficiency or cellulose accessibility on the hydrolysis. Therefore, the investigation of enzymatic cellulose hydrolysis should focus on changes in the cellulose polymer during the reaction in addition to soluble sugar analysis and cellulose molecular weight distributions should be measured standard-wise to correlate them to cellulose types and pretreatment methods.

Despite the advantages of cellulose gel permeation chromatography to analyze cellulose molecular weight distributions during enzymatic hydrolysis, only very few studies performed GPC measurements due to a complex and labor intensive sample preparation [9,10]. The primary challenge for chromatographic analysis is the very low solubility of cellulose. An established method to dissolve cellulose is the prior derivatization with phenylisocyanate to form tricarbanilates for dissolution in tetrahydrofuran [11]. Other well established methods dissolve the cellulose in dimethylacetamide/LiCl or 1,3-dimethyl-2-imidazolidinone/LiCl (DMI/LiCl) [12,13]. The latter methods have the advantage of analyzing non-derivatized cellulose but require swelling or activation of cellulose with ammonia or water before dissolution, or very long dissolution times [14,15]. As a result, the sample preparation for the analysis takes several days like for cellulose derivatization, significantly limiting the amount of samples that can be analyzed [16]. Therefore, new methods to prepare and analyze cellulose via GPC are highly desirable. One recent new approach has applied ionic liquids as solvent and eluent in cellulose analysis by GPC [17]. Ionic liquids are known to dissolve cellulose effectively [18]; but pose the disadvantage of high viscosity, requiring low flow rates of 0.01 mL/min that lead to impractically long analysis times. In conclusion, a viable and robust method that can analyze cellulose quickly and without extensive sample preparation is still missing.

In this work we present a new GPC method that does not require any cellulose activation or derivatization prior to cellulose analysis. The cellulose dissolution is based on a recent report using organic solvents containing small amounts of ionic liquid [19]. Here, we used a mixture of DMF and EMIM Ac to dissolve cellulose and to elute cellulose from the GPC column. The new solvent system was evaluated for the three commonly used cellulose types Avicel, α-cellulose and Sigmacell 101. As an application example, the effects of ionic liquid pretreatment on the cellulose molecular weight distributions during enzymatic hydrolysis were compared to that of native cellulose. Substantially altered cellulose molecular weight distributions during enzymatic hydrolysis suggest a different mode of action during enzymatic cellulose hydrolysis after ionic liquid pretreatment thus supporting the usefulness of cellulose molecular weight distribution analysis for biomass utilization.

## Results and discussion

### GPC analysis of commercial cellulose preparations in DMF/EMIM Ac

**Figure 1:**
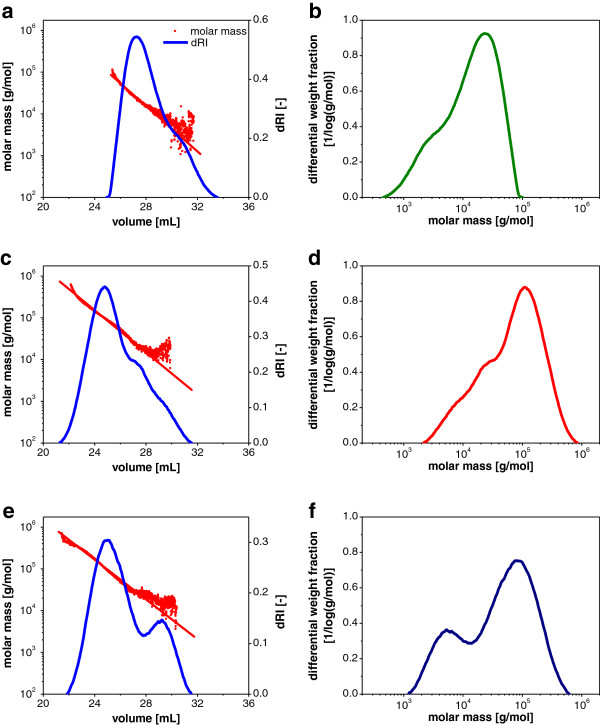
**GPC measurement of different cellulose types.** dRI and molecular mass elution profile for **(a)** Avicel, **(c)** α-cellulose, and **(e)** Sigmacell. Resulting differential weight distributions calculated for **(b)** Avicel, **(d)** α -cellulose and **(f)** Sigmacell. Eluent DMF/10% (v/v) EMIM Ac, 100 μL injection with 1 g/L cellulose and flow rate 0.5 mL/min.

To evaluate the new DMF/EMIM Ac solvent system for cellulose molecular weight distribution analysis by GPC, the three cellulose types Avicel, α-cellulose and Sigmacell were analyzed. MALLS measurement was used for absolute molecular weight determination because no ideal GPC standard for cellulose is available. In addition, MALLS measurement provides direct information on the quality of the separation method: A linear decrease of the logarithm of the molar mass with increasing elution volume indicates a pure size-based separation without stationary phase interaction. In Figure 1 the dRI and molar mass signals for the three cellulose types and the resulting differential molecular weight distributions are given.

Figure 1a, c and e illustrate the quality of the measurement with this new GPC method. Based on the dRI signal the mass recovery was calculated to be 102%, 99% and 109% for Avicel, α-cellulose and Sigmacell, respectively, indicating that cellulose was completely dissolved and detected in DMF/EMIM Ac with an acceptably low error. The dissolution of cellulose in DMF/EMIM Ac could be characterized further e.g. by nuclear magnetic resonance (NMR) or dynamic light scattering [20]. The mass recovery confirmed that the correct *dn/dc* value of 0.168 mL/g was used for this solvent system. The molecular weight signals of all three cellulose types showed a steadily declining molecular weight with increasing elution volume suggesting a truly size-based chromatographic separation. Beyond an elution volume of 30 mL lower molecular weight oligomers at relatively low concentrations eluted, resulting in increased noise of the light scattering signal due to the small refractive index increment. Nonetheless, all three cellulose types displayed the same slope of the log molar mass vs. elution volume regression, thereby indicating the high quality of the cellulose analysis. The differential molecular weight distributions were calculated using the corresponding parameters of the exponential fit lines.

From the differential molecular weight distributions in Figure 1b, d and f the weight average molecular weights *M*_*w*_ and the polydisperity, defined as *M*_*w*_*/M*_*n*_ (with *M*_*n*_ being the number average molecular weight), of the distributions were determined (Table 1). This confirmed the expected average molecular weight ranges to be the smallest for Avicel and the largest for α-cellulose. The measured *M*_*w*_ values for the different cellulose types were in the same order of magnitude and sequence as previous results [9,11,20-22]. However, comparing *M*_*w*_ values in the literature for the most defined cellulose, Avicel, shows variation between 0.49·10^4^ and 4.9·10^4^ g/mol [9,11,22]. This large variation can be attributed to a number of factors, i.e. lot-to-lot variability of the batches, different cellulose preparation methods, and most importantly different calibration standards used [11,16,20]. In particular, cellulose derivatization potentially results in a loss of lower molecular weight oligomers, leading to an overestimation of *M*_*w*_[9]. A possible derivatization of the reducing end of cellulose or degradation by EMIM Ac has been reported [23-25]. In this study, the cellulose degradation was minimized by applying moderate temperatures of < 80°C and short incubation times of 1 h for dissolution. A potential derivatization of cellulose at the reducing end would not significantly alter GPC analysis because the derivatized reducing end causes only negligible changes of the molecular weight of the high molecular weight polymer.

**Table 1:** Cellulose substrate characteristics

	***M***_***w***_**, g/mol**	**Polydispersity, -**	***CrI*, -**
Avicel PH 101	28,400	3.1	82%
Sigmacell 101	76,100	4.7	nd
α-cellulose	109,000	3	64%

Weight-average molecular weight and polydispersity was estimated from differential weight distributions shown in Figure 1 b, d, f. Crystallinity indices *CrI* are from Jäger et al. [20,26].

### Comparison of untreated and ionic liquid-pretreated and enzymatically hydrolysed commercial celluloses using GPC

**Figure 2:**
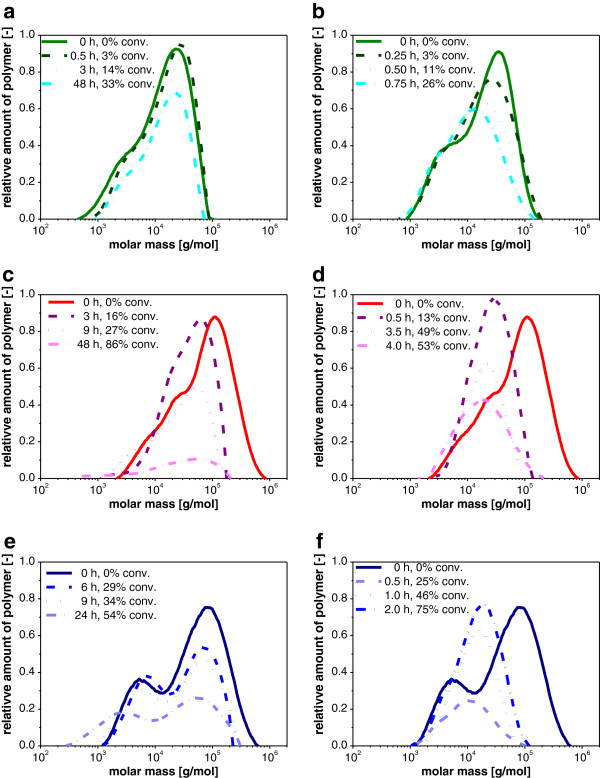
**Changes in differential weight distributions at different times during enzymatic hydrolysis. (a, c, e)** untreated and **(b, d, f)** regenerated cellulose using **(a, b)** Avicel, **(c, d)** α - cellulose **(e, f)** Sigmacell. Areas under the curves were adjusted to represent weight loss during the hydrolysis. Enzymatic hydrolysis performed at 45°C using 0.25 g/L desalted Celluclast® in 0.1 M NaAc buffer pH 4.8. Eluent DMF/10% (v/v) EMIM Ac, flow rate 0.5 mL/min.

After showing the general applicability of the new GPC method for cellulose analysis, the changes in the molecular weight distribution during enzymatic cellulose hydrolysis were investigated to understand how different cellulose properties and ionic liquid (IL) pretreatment affect the mechanism of the enzymatic hydrolysis. The differential molecular weight distributions during the hydrolysis of native and regenerated Avicel, α-cellulose and Sigmacell are compared in Figure 2.

The areas under the curves in Figure 2 correspond to the sugar based conversion of cellulose measured by high performance liquid chromatography (HPLC) and provided in the figure legend. During hydrolysis of untreated Avicel in Figure 2a the height of the distribution reduced along with the conversion. No significant change in the relative molecular weight distribution was observable up to the conversion of 33% reached after 48 h. Obviously, soluble sugars were produced almost exclusively from the cellulose polymer without affecting the relative molecular weight distribution. This result can be attributed to the structural properties of Avicel, which is microcrystalline cellulose with a crystallinity of 82% and a well-defined particle size of 44 μm on average [27,28]. Crystalline cellulose with a low accessible surface area is primarily hydrolyzed by exoglucanases that form cellobiose directly from cellulose [8,26,29]. Endoglucanases require a higher accessibility of the polymer. They hydrolyze only amorphous regions of cellulose in the interior of the polymer, resulting in reduced molecular weights [26]. Therefore, the results in Figure 2a demonstrate that endoglucanases play a minor role in the hydrolysis of untreated Avicel explaining the negligible shift in the molecular weight distributions at low conversion levels.

The first important observation in Figure 2b, d and f was that the hydrolysis reaction of regenerated cellulose proceeded drastically faster, which is in accordance with earlier results [30]. Furthermore, the ionic liquid pretreatment as it was performed here did not change the initial molecular weight distributions of the cellulose before the hydrolysis.

Other than the hydrolysis of untreated Avicel, the hydrolysis of Avicel regenerated from ionic liquids in Figure 2b resulted in a shift of the molecular weight distributions to lower molecular weights and a reduction of the *M*_*w*_ by 37% at a conversion of only 26%. Additionally, the height of the distribution reduced with increasing conversion, similar to that of untreated Avicel (Figure 2a). Differences in the hydrolysis of untreated and regenerated Avicel with the same cellulase mixture could be linked to the properties of regenerated cellulose: Regenerated cellulose is highly porous and amorphous, giving access to the individual cellulose polymer chains for the cellulases and also providing cleavage sites for endoglucanase [31]. Consequently, cellulose was now hydrolyzed in a synergistic manner, not only reducing the distributions in height but also shifting them to lower molecular weights. In summary, the cellulose pretreatment caused a shift of the hydrolysis mechanism from a merely exoglucanase activity to a combined exo- and endo-activity, resulting in stronger changes in the differential molecular weight distribution and additionally in a much faster conversion.

The enzymatic hydrolysis of untreated α-cellulose and Sigmacell is shown in Figure 2c and e, respectively. In contrast to untreated Avicel, a shift of the molecular weight distributions to lower molecular weights during enzymatic hydrolysis was observed for untreated α-cellulose and Sigmacell. In particular Sigmacell showed the strongest reduction in *M*_*w*_ by 43% from 7.61·10^4^ to 4.36·10^4^ g/mol at 54% conversion. Again, these results can be attributed to the structural properties of the two different cellulose types. Both were more amorphous than Avicel with a crystallinity of 64% for α-cellulose and no detectable crystallinity for Sigmacell [27,30]. Consequently, untreated α-cellulose and Sigmacell was more accessible to endo- and exoglucanases resulting in shifting differential molecular weight distributions. Nonetheless, even at high conversion there were still significant amounts of high molecular weight polymers remaining. E.g. after 48 h α-cellulose was converted to 86%, but contained still polymers larger than 2·10^5^ g/mol. Probably these were non-hydrolysable crystalline cellulose residuals.

The hydrolysis of regenerated α-cellulose and Sigmacell is shown in Figure 2d and f, respectively. Same as for regenerated Avicel, both substrates are amorphous, resulting in a shift of the differential molecular weight distributions to lower molecular weight during hydrolysis. For both substrates the *M*_*w*_ of the distribution strongly declined even at very low conversion: The *M*_*w*_ of regenerated α-cellulose reduced by 43% at less than 2% conversion. This was probably due to enhanced initial endoglucanase activity on the highly accessible regenerated cellulose and hints at a challenge in measuring the changes in the molecular weight distributions with high resolution at early incubation times: The hydrolysis reaction has to be quickly quenched to successfully ‘freeze’ the reaction progress. Otherwise, residual enzymatic activity will lead to further hydrolysis of the pre-treated substrate until analysis. In particular for pre-treated substrates where endo-attack of long polymer chains can easily occur, this leads to errors in the estimation of the *M*_*w*_.

**Figure 3:**
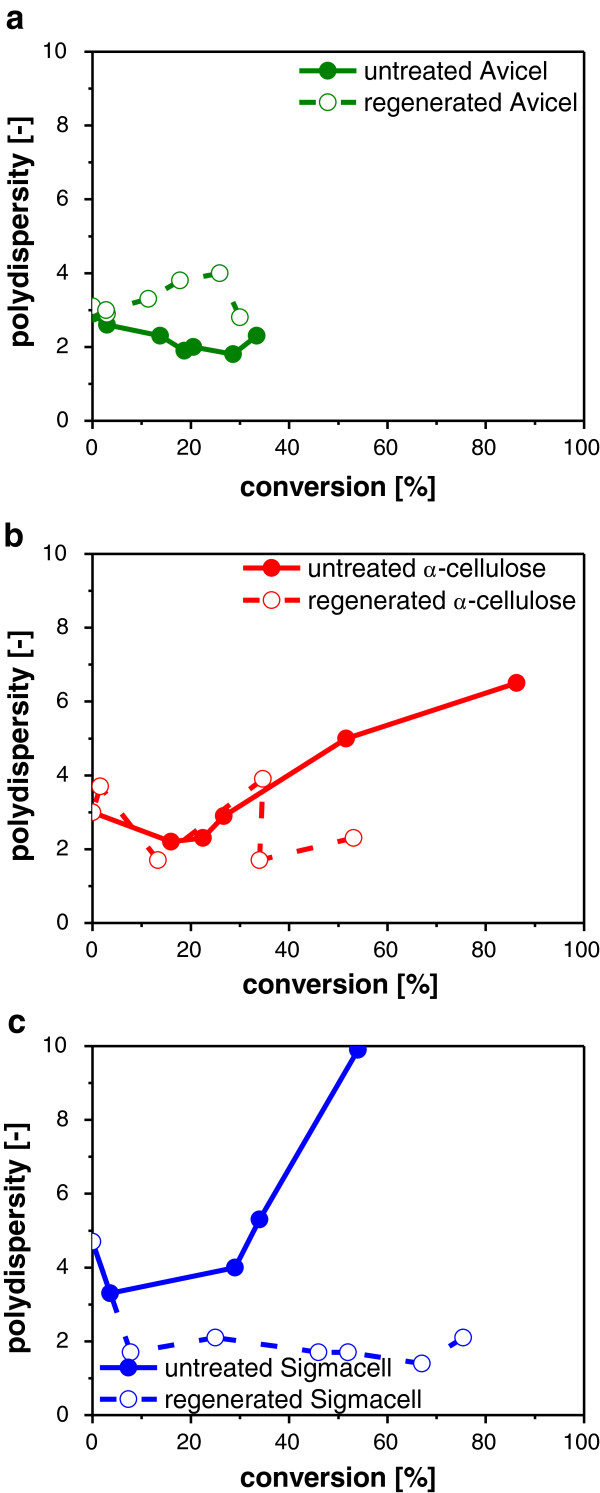
**Changes in polydispersity during enzymatic hydrolysis of untreated and regenerated cellulose. (a)** Avicel, **(b)** Sigmacell and **(c)** α-cellulose. Enzymatic hydrolysis performed at 45°C using 0.25 g/L desalted Celluclast® in 0.1 M NaAc buffer pH 4.8. Eluent DMF/10% (v/v) EMIM Ac, flow rate 0.5 mL/min.

In addition to the reduction in *M*_*w*_, also the shape of the molecular weight distribution changed during enzymatic hydrolysis (Figure 2). In particular, the differential molecular weight distributions of regenerated Sigmacell and α-cellulose and became narrower during hydrolysis. To illustrate the change of the distribution, the polydispersity during hydrolysis is depicted in Figure 3 for all untreated and regenerated celluloses.

The polydispersity of untreated cellulose increased substantially with increasing conversion except for Avicel that only reached very low conversion levels. An increasing polydispersity implies that the distribution becomes wider during hydrolysis: The number of smaller molecular weight polymers increased while the high molecular weight polymers were still present. In contrast to untreated cellulose, the polydispersity of regenerated α-cellulose and Sigmacell gradually declined with increasing conversion which is in accordance with the degradation of the high molecular weight polymers.

Previous studies that have investigated cellulose molecular weight distributions during hydrolysis include enzymatic hydrolysis [9], chemical hydrolysis [32-34] or both [10]. In summary it was observed that the cellulose molecular weight distributions changed much stronger for chemical hydrolysis than for enzymatic hydrolysis, which showed good agreement to our results. Chemical hydrolysis cleaves cellulose chains statistically reducing the polymer length at early reaction times. In contrast, cellulase mixtures are naturally evolved to convert cellulose effectively and selectively to soluble sugars for microbial uptake. The present study compared different cellulose types and the effect of ionic liquid pretreatment on enzymatic hydrolysis for the first time. In future, the combination of sugar measurements and cellulose polymer molecular weight distributions will contribute fundamentally to understand enzymatic cellulose hydrolysis, to evaluate pretreatment strategies, and to design optimal cellulase mixtures.

## Conclusions

We presented a new chromatographic method for cellulose GPC analysis employing DMF/EMIM Ac as cellulose solvent and eluent that eliminates time intensive sample preparation and allows to measure larger sample numbers necessary for in-depth understanding of enzymatic cellulose hydrolysis. This method can potentially be extended for the measurement of lignin and wood, similar to earlier approaches working with ionic liquids [21,35]. If the analysis of the cellulose molecular weight distributions will be extended from the pure qualitative evaluation performed in this study to a quantitative analysis and interpretation using mechanistically sound mathematical models of enzymatic cellulose degradation, this novel GPC analysis will be able to play a key role in building a holistic understanding of enzymatic cellulose hydrolysis.

## Methods

### Materials

The cellulose substrates Avicel PH101, Sigmacell 101, and α-cellulose were purchased from Sigma-Aldrich (St Louis, USA). The ionic liquid 1-ethyl-3-methylimidazolium acetate (EMIM Ac) was kindly provided by BASF (Ludwigshafen, Germany). The commercial cellulase preparation Celluclast® 1.5 L from *T. reesei* ATCC26621 (Novozyme, Denmark) with an activity of 700 EGU/g according to manufacture specification was used in the hydrolysis experiments. Prior to cellulose hydrolysis, the cellulase was rebuffered in 0.1 M sodium acetate buffer pH 4.8 with a HiPrep 26/10 desalting column (GE Healthcare, Buckinghamshire, Great Britain) using an ÄKTA FPLC system (GE Healthcare) [28]. The rebuffered Celluclast® had a specific activity of 244 U/g according to the standard filter paper assay [26,36].

### Sample preparation

Dry cellulose was dissolved for the GPC measurement without any prior swelling, activation, or derivatization. 1–2 g/L cellulose was dissolved in dimethylformamide (DMF) containing 10 - 20% (v/v) EMIM Ac at 80°C for 1 h. 2 g/L Avicel dissolved almost instantaneously in DMF containing 10% (v/v) EMIM Ac and formed a visually clear solution. 1 g/L α-cellulose and Sigmacell 101 in DMF containing 20% (v/v) EMIM Ac formed a clear solution within less than 10 minutes. The volume percentage EMIM Ac required for dissolution should generally increase with degree of polymerisation and concentration of cellulose to be dissolved. For measurement of cellulose during enzymatic hydrolysis, 1 mL samples, containing 10 mg cellulose were dried over night at 80°C prior to the dissolution of 1 mg dried cellulose in 1 mL DMF/EMIM Ac. Before injection, the samples were filtered with a Whatman 1 μm polytetrafluorethylene (PTFE) filter (GE Healthcare).

### Gel permeation chromatography

The gel permeation chromatography system consisted of an isocratic pump, auto-sampler with thermostat (Agilent 1200 series, Santa Clara, USA); and a set of GRAM separation columns, one 30 Å and two 10 000 Å columns (PSS, Mainz, Germany) kept at 60°C in a column oven (K7, Techlab, Erkerode, Germany). A MALLS detector was used for absolute molecular weight measurement (DAWN HELEOS 8+ λ 658 nm, Wyatt Technologies, Santa Barbara, USA) and a differential refractive index (dRI) detector (Optilab rEX λ 658 nm, Wyatt Technologies) for quantification. Data acquisition of molecular weight was performed with Astra Software (Wyatt Technologies) using Zimm first order as model. 100 μL samples with 1–2 g/L cellulose in DMF containing 10-20% (v/v) EMIM Ac were injected and analyzed at a flow rate of 0.5 mL/min DMF containing 10% (v/v) EMIM Ac as eluent. Linear regression of log MW vs. retention time resulted in R^2^ larger than 0.9 for all samples. Replicate samples resulted in deviations of < 10% with respect to *M*_*W*_ and *M*_*n*_, respectively.

### Measurement of specific refractive index increment

The refractive index increment (*dn/dc*) for cellulose in DMF/10% (v/v) EMIM Ac was determined using Optilab rEX dRI detector. Ten 20-100 μL samples of 0.5 g/L cellulose in DMF/10% (v/v) EMIM Ac were directly injected into the dRI detector. From the peak in the dRI detector the *dn/dc* value was then calculated based on 100% mass recovery in the ASTRA software. The resulting *dn/dc* value was 0.168±0.006 mL/g. This value was further confirmed by the GPC measurements of the cellulose, showing a mass recovery in the dRI signal of 95-110% in all measurements.

### Cellulose pretreatment

The regenerated cellulose from ionic liquid pretreatment was obtained by dissolving 50 g/L of the respective cellulose in EMIM Ac at 80°C in a thermo mixer MHR23 (HLC Biotech, Bovenden, Germany) for 1 h until a clear solution was formed. The cellulose was precipitated by 0.1 M sodium acetate buffer pH 4.8 while vigorously agitating. The regenerated cellulose was washed subsequently with 100 fold buffer volume to remove residual ionic liquid. The regenerated cellulose was kept wet at 4-8°C until use. The dry mass fraction of the regenerated cellulose was obtained by drying a cellulose sample at 104°C over night. The dry mass fraction of the different cellulose types were determined to 8.3% 10.4% and 9% for Avicel, α-cellulose, and Sigmacell, respectively.

### Enzymatic hydrolysis

Cellulose hydrolysis was performed at 45°C in 0.1 M sodium acetate buffer pH 4.8 with 10 g/L cellulose and 0.25 g/L desalted cellulase in 15 mL Falcon tubes in a MHR 23 (HLC Biotech, Bovenden, Germany). To stop the enzymatic hydrolysis, the samples were transferred to an ice bath for 20 min. The undissolved cellulose was afterwards removed by centrifugation at 4°C. The glucose and cellobiose concentration in the supernatant were determined by Dionex HPLC with refractive index detector (STH 585, 232 XL, E580) using an 300x4 mm organic acid column (CS-Chromatographie, Langerwehe, Germany) with a flow rate of 1 mL/min and 5 mM H_2_SO_4_ as eluent. The undissolved cellulose pellet was further inactivated at 100°C in a water bath for 10 min and afterwards dried at 85°C over night before the GPC measurement.

## Abbreviations

DMF: DiMethylFormamide; dRI: Differential refractive index; EMIM Ac: 1-Ethyl-3-Methyl-lmidazolium acetate; GPC: Gel permeation chromatography; HPLC: High performance liquid chromatography; IL: Ionic liquid; MALLS: Multi angle laser light scattering; NMR: Nuclear magnetic resonance; PTFE: PolyTetraFluorEthylene.

## Competing interests

The authors declare no competing interests.

## Authors’ contributions

PE designed the experiments, LH and PE performed the cellulose sample preparations and analyses as well as the enzymatic reactions. PE analysed the data and drafted the manuscript. AS conceived the study and helped to draft the manuscript. All authors read and approved the final manuscript.
